# Specificity of presenilin‐1‐ and presenilin‐2‐dependent γ‐secretases towards substrate processing

**DOI:** 10.1111/jcmm.13364

**Published:** 2017-10-10

**Authors:** Serena Stanga, Céline Vrancx, Bernadette Tasiaux, Claudia Marinangeli, Helena Karlström, Pascal Kienlen‐Campard

**Affiliations:** ^1^ Alzheimer Research group Institute of Neuroscience Université catholique de Louvain Brussels Belgium; ^2^ Inserm CHU Lille UMR‐S 1172 – JPArc – Centre de Recherche Jean‐Pierre AUBERT University of Lille Lille France; ^3^ Center for Alzheimer Research Division of Neurogeriatrics Department of Neurobiology, Care Sciences and Society Karolinska Institute Stockholm Sweden

**Keywords:** presenilins, γ‐secretase complex, APP, Notch, reporter gene assay, γ‐secretase inhibitors, Alzheimer's disease

## Abstract

The two presenilin‐1 (PS1) and presenilin‐2 (PS2) homologs are the catalytic core of the γ‐secretase complex, which has a major role in cell fate decision and Alzheimer's disease (AD) progression. Understanding the precise contribution of PS1‐ and PS2‐dependent γ‐secretases to the production of β‐amyloid peptide (Aβ) from amyloid precursor protein (APP) remains an important challenge to design molecules efficiently modulating Aβ release without affecting the processing of other γ‐secretase substrates. To that end, we studied PS1‐ and PS2‐dependent substrate processing in murine cells lacking presenilins (PSs) (PS1KO, PS2KO or PS1‐PS2 double‐KO noted PSdKO) or stably re‐expressing human PS1 or PS2 in an endogenous PS‐null (PSdKO) background. We characterized the processing of APP and Notch on both endogenous and exogenous substrates, and we investigated the effect of pharmacological inhibitors targeting the PSs activity (DAPT and L‐685,458). We found that murine PS1 γ‐secretase plays a predominant role in APP and Notch processing when compared to murine PS2 γ‐secretase. The inhibitors blocked more efficiently murine PS2‐ than murine PS1‐dependent processing. Human PSs, especially human PS1, expression in a PS‐null background efficiently restored APP and Notch processing. Strikingly, and contrary to the results obtained on murine PSs, pharmacological inhibitors appear to preferentially target human PS1‐ than human PS2‐dependent γ‐secretase activity.

## Introduction

Presenilins 1 and 2 are homologous polytopic proteins that represent the catalytic subunits of the γ‐secretase complex. The assembly and activation of the γ‐secretase is achieved by the association of either PS1 or PS2 1 with three other membrane proteins: nicastrin (Nct), anterior pharynx defective 1 (Aph1a or Aph1b in humans; Aph1a, Aph1b or Aph1c in rodents 2) and presenilin enhancer 2 (Pen‐2), giving rise to four and six different γ‐secretases in humans and rodents, respectively. The γ‐secretase is responsible for the cleavage of up to 90 type I integral membrane proteins 3, 4 that have previously undergone an ectodomain shedding by processing at a cleavage site close to their transmembrane domain 5. This results in the generation of a membrane‐anchored C‐terminal fragment (CTF), which is subsequently cleaved by the γ‐secretase within its transmembrane domain to generate a truncated membrane stub and a soluble intracellular domain (ICD). The APP and the Notch receptor are the predominant substrates of γ‐secretase (PSs) 6. Their processing is of critical importance in AD aetiology and in cell proliferation, development and tissue homoeostasis. The onset and progression of AD relies on the release of various Aβ generated by the γ‐secretase cleavage of APP 7. Aβ is the main component of the senile plaques found in brains of AD patients 8. Notch signalling is triggered by the γ‐secretase release of its intracellular domain (NICD) that exerts essential functions during development 9.

Much attention in AD research has focused on the γ‐secretase‐mediated cleavage of the amyloidogenic CTF of APP (β‐CTF or C99) that generates the intracellular domain of the protein (AICD) and the Aβ peptide 10. To block amyloid deposition and the formation of senile plaques, many pharmacological approaches have been designed to target the γ‐secretase activity, but clinical trials of γ‐secretase inhibitors or modulators have been halted due to severe adverse effects 11. These adverse effects were primarily due to the concomitant inhibition of the γ‐secretase‐dependent cleavage of Notch (NotchΔE) 12 producing NICD, thus precluding its signalling critical in cell fate decision/cell regeneration in the adult organism 13. To note, Notch protein also acts as a tumour suppressor, leading to an increased risk of cancers when its processing is inhibited 14, in accordance with adverse effects observed in clinical trials testing γ‐secretase inhibitors.

In this context, our primary goal was to investigate the respective contribution of PS1‐ and PS2‐dependent γ‐secretases in the cleavage of their principal substrates, APP and Notch. Discrimination of PS1 and PS2 γ‐secretases activities has remained elusive but has gained considerable interest with very recent studies showing that PS1 and PS2 γ‐secretases can be discriminated by their subcellular localization, with an impact on substrate processing 15, 16. However, the understanding of substrate specificities of PS1 and PS2 γ‐secretases and their functional relevance remains very limited. To investigate the precise contribution of PS1 and PS2 to APP and Notch processing, we used a homologous approach 17 allowing to directly compare the processing of APP and Notch by a reporter assay measuring the release of AICD and NICD. The transactivation of reporter genes is achieved by the presence of a Gal4‐VP16 sequence fused to AICD or NICD, efficiently activating a response element Gal4RE coupled to a luciferase reporter gene. These tags (Gal4‐VP16) were previously shown not to interfere with APP and Notch processing and to allow a highly specific and sensitive detection of the processing of both substrates by γ‐secretase 17, granting the comparison of the two cleavages in a standardized system. We carried out our study in mouse embryonic fibroblasts (MEFs) either expressing both PSs (PS wild‐type; PS1+/+PS2+/+ noted PS+/+), none of them (PS double‐knockout; PS1−/−PS2−/− referred to as PSdKO) or only one (PS1 or PS2 single‐KO; PS1−/−PS2+/+ and PS1+/+PS2−/− referred to as PS1KO and PS2KO, respectively). We also used cells stably expressing human PS1 or PS2 (rPS1wt, rPS2wt) in an endogenous PS‐null background (PSdKO) as well as cells stably expressing catalytically inactive forms of human PS1 or PS2 (rPS1DA, rPS2DA) in the same background 18. We tested in our models two well‐characterized pharmacological γ‐secretase inhibitors: DAPT 19 and L‐685,458 20 (noted L‐685), to study their effects on PS1‐ and/or PS2‐dependent processing of APP and Notch.

Our results show that murine (endogenous) PS1, but not PS2, is strongly involved in the processing of APP and Notch. Absence of PS1 (PS1KO) provokes a drastic reduction of APP and Notch cleavages, which are restored by the expression of human PS1 (rPS1wt). These observations are consistent with previously published data 21, 22 underlining the predominant role of PS1 in γ‐secretase‐dependent substrate processing. Strikingly, our results further indicate that pharmacological γ‐secretase inhibitors differently affect PS1 and PS2 γ‐secretases. More precisely, endogenous murine PS1 (in PS2KO) appears resistant to the pharmacological inhibition while endogenous murine PS2 (in PS1KO) is sensitive to it. On the contrary, human PS1 is more affected by inhibitory treatments than its homolog PS2 (in rPS1wt and rPS2wt, respectively). Finally, the results obtained with the catalytically inactive human PSs (rPSDA) suggest that PS2 may have a role in APP cleavage independently of its enzymatic activity, whereas PS1‐dependent cleavage requires a native catalytic site.

## Materials and methods

### Cell lines and cell culture

Mouse embryonic fibroblasts (MEFs) wild‐type (PS+/+) and knockout cell lines for PS1 (PS1KO), PS2 (PS2KO) or both (PSdKO) were a kind gift of B. De Strooper (KUL, Leuven, Belgium). Original plasmids expressing wild‐type or mutant forms of human PS1 and PS2 and lentiviral constructs used to produce stable rescued cell lines from MEFs PSdKO (rPS) were described previously 18. MEFs cells were cultured in DMEM‐F12 media (Life Technologies, Carlsbad, CA, USA). All media were supplemented with 10% of foetal bovine serum (FBS) (Thermo Scientific, Rockford, IL, USA) and penicillin–streptomycin solution (10 units–10 μg). Puromycin (2.5 μg/ml) was used for the selection and maintenance of rPS cells. All cell cultures were maintained at 37°C in a humidified atmosphere (5% CO_2_).

### Chemicals and reagents

Reagents for Western blotting and BCA protein assay kit were from Pierce (Rockford, IL, USA), membranes and ECL+ from GE Healthcare (Little Chalfont, UK). Primary antibodies anti‐PS1 and anti‐PS2 were from Cell Signaling (Danvers, MA, USA), anti‐VP16 and anti‐Notch1 were from Abcam (Cambridge, UK), and anti‐actin and anti‐tubulin antibodies and the fluorescent nucleic acid stain DAPI were obtained from Sigma‐Aldrich (St Louis, MO, USA). Primary antibody directed against APP‐CTF was kindly received from N. Sergeant (INSERM, Lille, France). Secondary antibodies coupled to peroxidase were obtained from Amersham Bioscience (Uppsala, Sweden). The applied dilutions for each antibody were according to the manufacturer's instructions. Alexa‐labelled secondary antibodies were obtained from Life Technologies. Fluorescent mounting medium was from DAKO (Agilent Technologies, Santa Clara, CA, USA). TRIzol reagent and Complete^TM^ protease inhibitor cocktail were from Roche (Basel, Switzerland). The cDNA synthesis kit and iQ SYBR Green Supermix were from Bio‐Rad (Hercules, CA, USA).

### Western blotting

Cells were rinsed and scraped off in PBS and centrifuged for 5 min. at 7.000 g. Pellets were sonicated in lysis buffer (125 mM Tris pH 6.8, 20% glycerol, 4% SDS) with Complete™ protease inhibitor cocktail. Protein concentration was determined using the BCA protein assay kit. Fifteen to forty micrograms of protein were heated for 10 min. at 70°C in loading buffer (lysis buffer containing 0.5 M DTT and staining SeeBlue^®^ Plus2, Life Technologies), loaded and separated onto 4–12% Nupage™ bis‐Tris gels (Life Technologies), and then transferred for 2 hrs at 30V onto nitrocellulose membranes. After blocking (5% non‐fat milk in PBS), membranes were incubated overnight at 4°C with the primary antibodies, then washed, and incubated with the secondary antibodies coupled to peroxidase prior to ECL detection. Primary antibodies are as follows anti‐PS1 (1:1.000), anti‐PS2 (1:1.000), anti‐VP16 (1:500), anti‐Notch1 (1:500), anti‐APP‐CTF (1:5000), anti‐actin (1:3000) or anti‐tubulin (1:3000). Secondary antibodies are as follows anti‐mouse (1:10.000) or anti‐rabbit (1:10.000).

### RNA preparation, qPCR and RT‐PCR

The RNAs were extracted from cells and tissues in TRIzol reagent and reverse‐transcribed using an iScript cDNA synthesis kit. Real‐time qPCR analyses were performed on 2 ng of cDNA template using iQ SYBR Green Supermix in an iCycler IQ multicolour Real‐Time PCR detection system. qPCR conditions were typically 95°C for 30 sec., followed by 40 cycles of 30 sec. at 95°C, 45 sec. at 60°C and 15 sec. at 79°C and ended by 71 cycles of 30 sec. at 60°C. The relative changes in the target gene‐to‐GAPDH mRNA ratio were determined by the 2^(−ΔΔCt)^ calculation. The sequences for qPCR primers are as follows:

**Table:** 

PS1	Forward	5′‐GCCACCATCAAATCAGTCAG‐3′
	Reverse	5′‐ATACAGGACCACCAGGAGG‐3′
PS2	Forward	5′‐GCGAAGAAGACTGTGAAGAG‐3′
	Reverse	5′‐AGAAACGCACAGACTTGATAG‐3′
GAPDH	Forward	5′‐ACCCAGAAGACTGTGGATGG‐3′
	Reverse	5′‐ACACATTGGGGGTAGGAACA‐3′

### Immunocytochemistry

Cells were seeded and transfected on 12‐well plates. Forty eight hours after transfection, cells were rinsed twice with Opti‐MEM and fixed with 4% paraformaldehyde (PFA) for 15 min. After three washes in PBS, cells were permeabilized with PBS1X/0.3% Triton 100‐X for 30 min. and blocked in PBS1X/FBS5%/0.1% Triton 100‐X for 30 min. Primary antibodies (anti‐PS1, PS2 and VP16) were prepared in the blocking solution (1:100) and incubated O/N at 4°C. After three washes in PBS, cells were incubated with secondary antibody (goat antimouse Alexa 594 and goat antimouse Alexa 488, 1:500 in blocking solution) and DAPI (1:2000) for 1 hr at 4°C. After three washes in PBS, cells were stored in PBS‐azide 0.1% at 4°C. Pictures were taken with an Olympus Fluoview confocal microscope (Olympus America Inc., Center Valley, PA, USA).

### DNA constructs

The pCDNA3.1 plasmid bearing the C99‐GVP construct, the pCS2 harbouring NotchΔE‐GVP, the Gal4RE Firefly luciferase reporter gene (pG5E1B‐luc) and the Renilla luciferase reporter vector (phRG‐TK) have been previously described 17, 23.

### Cell transfection

MEFs were transfected 24 hrs after seeding using Trans‐IT2020 (Mirus Bio Corporation, Madison, WI, USA). Transfection was performed according to the manufacturer's instructions. Cell lysates were harvested 48 hrs after transfection for analysis.

### Pharmacological treatments

Two specific γ‐secretase inhibitors were used: *N*‐[*N*‐(3,5‐difluorophenacetyl)‐L‐alanyl]‐sphenylglycine *t*‐butyl ester or DAPT (Calbiochem, Camarillo, CA, USA), and L‐685,458 (Sigma‐Aldrich), noted L‐685. MEFs cells were treated with 0.25, 1 or 10 μM of DAPT and with 10 or 20 μM of L‐685. Cells were harvested 16 hrs after pharmacological treatments for analysis.

### Luciferase

Cells were cotransfected in a 1:1:1 ratio with pG5E1B‐luc, phRG‐TK and either pCDNA3.1‐C99‐GVP or pCS2‐NotchΔE‐GVP to quantify the release of AICD‐GVP or NICD‐GVP, respectively. Forty eight hours after transfection, cells were rinsed with PBS and incubated with the reporter lysis buffer (Promega, Madison, WI, USA) for 15 min. at room temperature. Firefly and Renilla luciferase activities were measured using the Dual‐Glo^®^ Luciferase Assay System on a Sirius luminometer (Berthold, Pforzheim, Germany). Luciferase activity corrected for transfection efficiencies was calculated as the Firefly/Renilla luciferase ratio.

### Statistical analysis

The number of samples (*n*) in each experimental condition is indicated in the figure legends. Statistical analyses were performed using GraphPad Prism software (GraphPad Software, La Jolla, CA) by ANOVA followed by Bonferroni's multiple comparison tests.

## Results

### Endogenous murine PS1 and PS2 involvement in C99‐GVP and Notch∆E‐GVP cleavage

**Figure 1:**
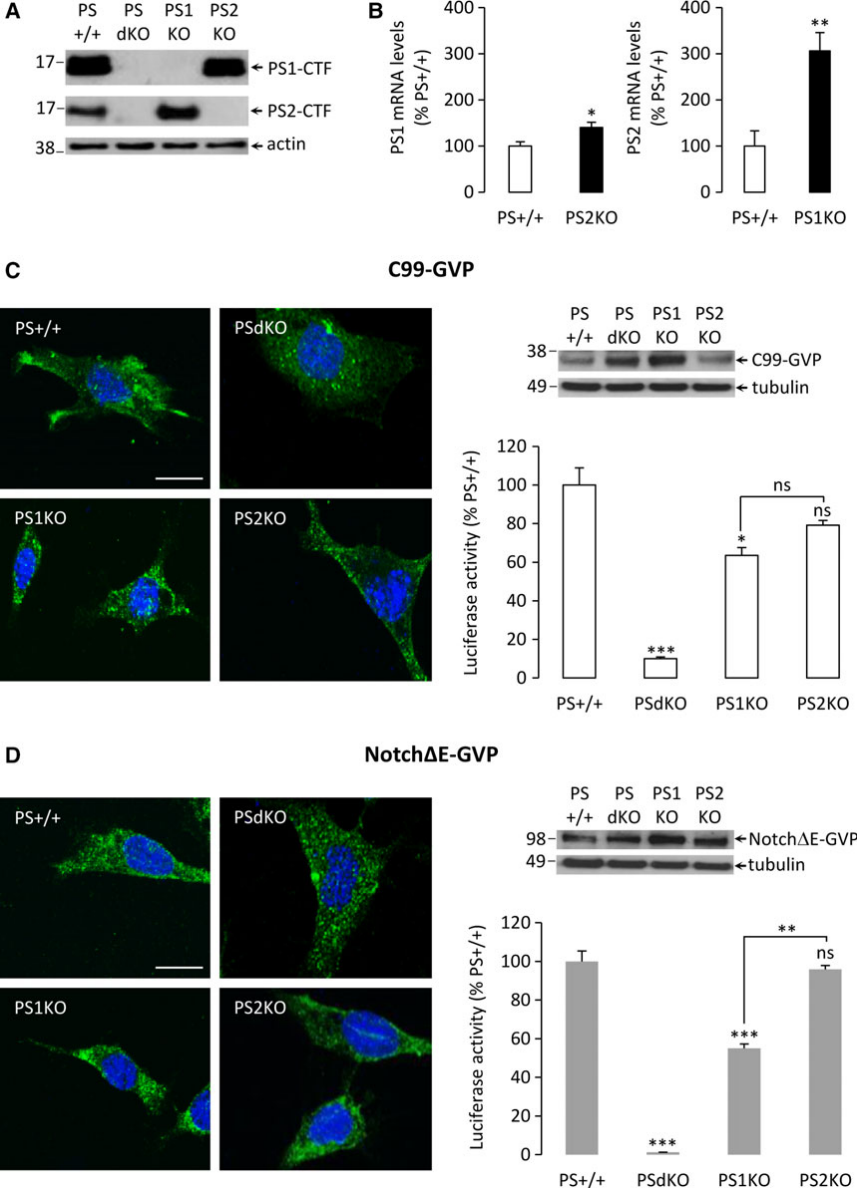
Characterization of PS wild‐type, single and double‐knockout MEFs cell lines. (**A**) Western blot of cell lysates from PS+/+, PSdKO, PS1KO and PS2KO MEFs revealed with anti‐PS1‐CTF and anti‐PS2‐CTF antibodies. Actin was used as a protein loading control. Results exhibited the presence of endogenous PS1‐CTF in PS2KO, and of PS2‐CTF in PS1KO cells. (**B**) qPCR quantification of endogenous PS1 and PS2 mRNA levels in wild‐type and single‐KO conditions. Results (means ± S.E.M.) are represented as percentages of PS+/+ signals. *P < 0.05, **P < 0.01 *versus* PS+/+, *n* = 5. (**C**) Immunocytochemistry (left panel) was performed in PS+/+, PSdKO, PS1KO and PS2KO MEFs transfected with the C99‐GVP construct. Cells were immuno‐stained for VP16‐tag (green) and DAPI (blue). Stainings have been analysed by confocal microscopy to generate *z*‐stack images. Western blotting (right panel, top) was performed with an antibody directed against the VP16 domain of the GVP fusion protein to check the transfection efficiency of C99‐GVP construct, and quantification of AICD‐GVP release (right panel, bottom) was monitored by luciferase activity. Results (means ± S.E.M.) are represented as percentages of PS+/+ signals. *P < 0.05, ***P < 0.001 *versus* PS+/+, *n* = 9 in three independent experiments. (**D**) Left, immunocytochemistry was performed in PS+/+, PSdKO, PS1KO and PS2KO MEFs transfected with the NotchΔE‐GVP construct and analysed as described in (**C**). Right, Western blotting with an antibody directed against VP16 domain of the GVP fusion protein (top) and quantification of NICD‐GVP release (bottom). Results (means ± S.E.M.) are represented as percentages of PS+/+ signals. **P < 0.01, ***P < 0.001 *versus* PS+/+, *n* = 9 in three independent experiments. Scale bar: 20 μm.

The PSs expression profile was monitored in each MEFs cell line used for this study; PS+/+, PSdKO, PS1KO and PS2KO by Western blotting with antibodies directed against the CTF of PS1 or PS2 (Fig. 1A). It appeared that absence of a PS can be compensated by an increased expression of the other, especially in the case of PS2 levels increased in the absence of PS1. This was confirmed by qPCR, showing that PS2 mRNA levels are higher (+200%) in PS1KO than in PS+/+ cells (Fig. 1B). Interestingly, absence of PS2 had only moderate effects on PS1 transcription (+40%). Cross‐regulation of PSs expression has already been reported 24 and must be kept in mind here for the interpretation of the respective contribution of PS1 and PS2 to APP and Notch processing.

To investigate the particular role of PS1 and PS2 in substrate cleavage, we expressed C99‐GVP (Fig. 1C) and NotchΔE‐GVP (Fig. 1D) constructs in our MEFs. These constructs have been developed to allow direct comparison of APP and Notch processing in a defined cellular context 17. The release of APP and Notch intracellular domains (AICD and NICD, respectively) from exogenous substrates (C99‐GVP and NotchΔE‐GVP) was measured by a Gal4 reporter gene assay. We first verified by immunocytochemistry using an antibody directed against the VP16 domain of the GVP fusion protein that they were properly expressed and localized in the different cell lines. Results showed that the fusion proteins were efficiently expressed with a consistent subcellular localization in the cells (Fig. 1C and D). Moreover, Western blotting with the same antibody directed against VP16 showed that the transfection efficiency of both C99‐GVP and NotchΔE‐GVP constructs was comparable in all cell types (Fig. 1C and D). The specificity of the signals detected around 35 kDa and 100 kDa for C99‐GVP and NotchΔE‐GVP, respectively, was checked by comparison of non‐transfected *versus* transfected cells (Fig. S1).

Luciferase assays indicated that the γ‐secretase cleavages of both C99‐GVP and NotchΔE‐GVP were abolished in PSdKO, as expected. To note, a low residual activity was measured for C99‐GVP in PSdKO cells, likely due to a basal leaking of C99 constructs in the nuclear compartment (Fig. 1C). We observed a significant decrease in the luciferase activity especially for Notch∆E‐GVP in PS1KO cells, while in PS2KO cells, the signals were not significantly different from those measured in PS+/+ cells. This indicates that endogenous murine PS1 is predominantly involved in AICD‐GVP and NICD‐GVP production.

### Rescue of APP and Notch cleavage by re‐expression of human catalytically active PSs in a PS‐null background

**Figure 2:**
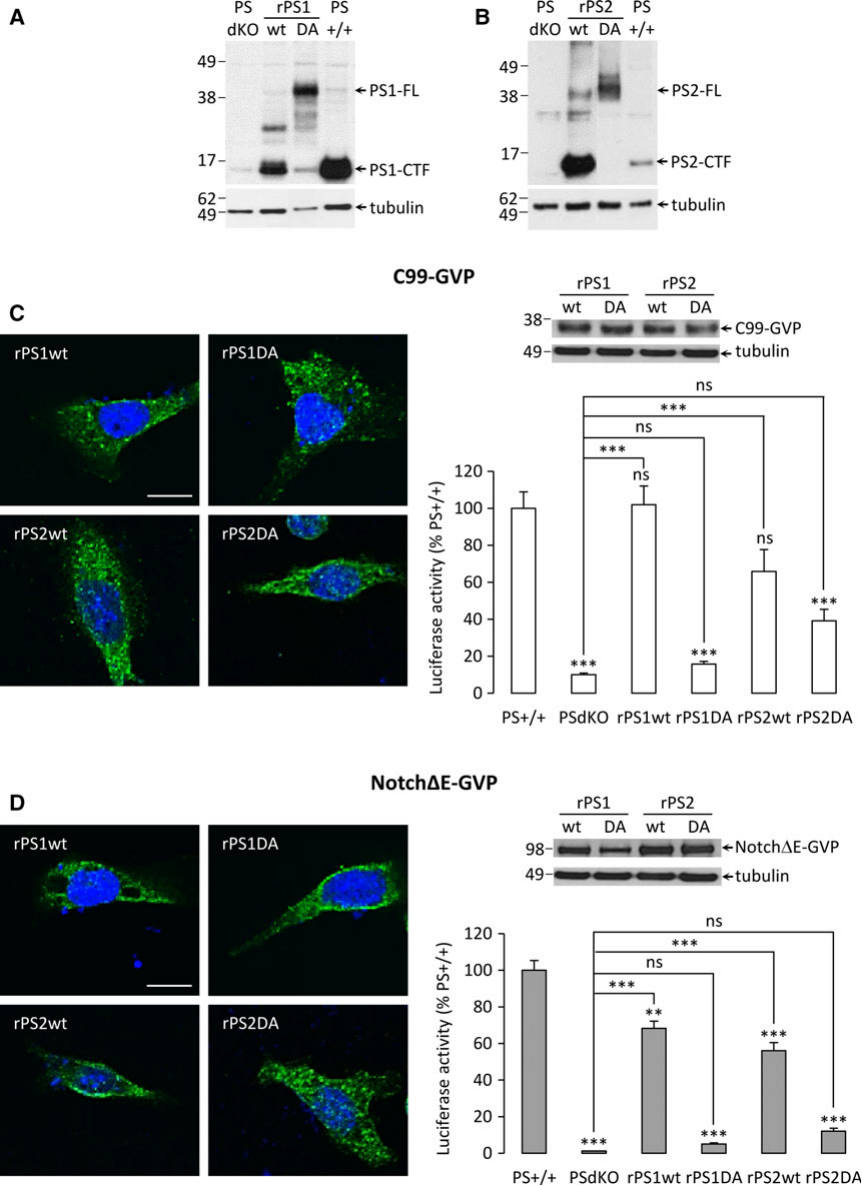
Characterization of rescued cell lines expressing human PSs. (**A**) Cell lysates from PS+/+, PSdKO, rPS1wt and rPS1DA MEFs were analysed by Western blotting. The presence of PS1, full‐length and CTF, was detected by the anti‐PS1‐CTF antibody. Tubulin was used as a protein loading control. Results exhibited the restauration of full‐length PS1 expression in both rescued models, with an active form (PS1‐CTF) only in rPS1wt. (**B**) Cell lysates from PS+/+, PSdKO, rPS2wt and rPS2DA MEFs were analysed by Western blotting for the presence of PS2, full‐length and CTF, using the anti‐PS2‐CTF antibody. Tubulin was used as a protein loading control. Results exhibited the restauration of PS2‐full‐length expression in both rescued models, with an active form (PS2‐CTF) only in rPS2wt. (**C**) Immunocytochemistry was performed in rPS1wt, rPS1DA, rPS2wt and rPS2DA MEFs transfected with the C99‐GVP construct (left panel). Cells were immuno‐stained for VP16‐tag (green) and DAPI (blue). Stainings have been analysed by confocal microscopy to generate *z*‐stack images. Western blotting (right panel, top) was performed with an antibody directed against the VP16 domain of the GVP fusion protein to check the transfection efficiency of C99‐GVP construct, and quantification of AICD‐GVP release (right panel, bottom) was monitored by luciferase activity. Results (means ± S.E.M.) are represented as percentages of PS+/+ signals. ***P < 0.001 *versus* PS+/+, *n* = 9 in three independent experiments. (**D**) Immunocytochemistry was performed in rPS1wt, rPS1DA, rPS2wt and rPS2DA MEFs transfected with the NotchΔE‐GVP construct (left panel) and analysed as described in (**C**). Right, Western blotting with the VP16 antibody (top) and quantification of NICD‐GVP release (bottom). Results (means ± S.E.M.) are represented as percentages of PS+/+ signals. **P < 0.01, ***P < 0.001 *versus* PS+/+, *n* = 9 in three independent experiments. Scale bar: 20 μm.

Mouse embryonic fibroblasts stably re‐expressing human wild‐type or catalytically inactive PSs (rPS1/2wt, rPS1/2DA) in a PSdKO background have been previously described 18. We monitored human PSs expression and activation by Western blotting (Fig. 2A and B) in these cells. Both PS1 and PS2‐CTFs were detected in PS+/+, as well as in rPS1wt and rPS2wt cells, respectively. These CTFs were absent in the DA mutants, where an accumulation of full‐length PSs (around 45 kDa) is observed. This is characteristic of catalytically inactive forms of PSs that do not undergo endoproteolytic activation. The expression of both C99‐GVP and NotchΔE‐GVP constructs was verified in rescued cell lines using immunocytochemistry with the antibody directed against the VP16 domain of the GVP fusion protein. Both fusion proteins were efficiently expressed with a consistent subcellular localization in the cells (Fig. 2C and D). Western blotting showed that the transfection efficiency of both C99‐GVP and NotchΔE‐GVP constructs was comparable in all cell types (Fig. 2C and D).

We measured the effect of PSs re‐expression on C99‐GVP and Notch∆E‐GVP processing by γ‐secretase (Fig. 2C and D). C99‐GVP cleavage observed in rPS1wt was comparable to the one measured in PS+/+ cells (Fig. 2C). In rPS2wt cells, the restoration appears less efficient but the signal triggered by C99‐GVP cleavage is not significantly different from PS+/+ cells (Fig. 2C). Notch∆E‐GVP processing was lower in PSs rescued cells when compared to PS+/+ cells, although significantly restored when compared to the PSdKO control cells (Fig. 2D). To note, the levels of human PS2 expression, higher than endogenous PS2 levels, might lead to an overestimation of the PS2‐dependent γ‐secretase activity in the PS2‐rescued cells (Fig. 2A). The inactivation of PS1 catalytic activity (rPS1DA) abolished the cleavage of both substrates (Fig. 2C and D). In rPS2DA cells, a residual luciferase activity was measured in C99‐GVP expressing cells but it is non‐significant when compared to PSdKO controls.

### Effects of γ‐secretase inhibitors on APP and Notch processing in PS1 or PS2 expressing cells

**Figure 3:**
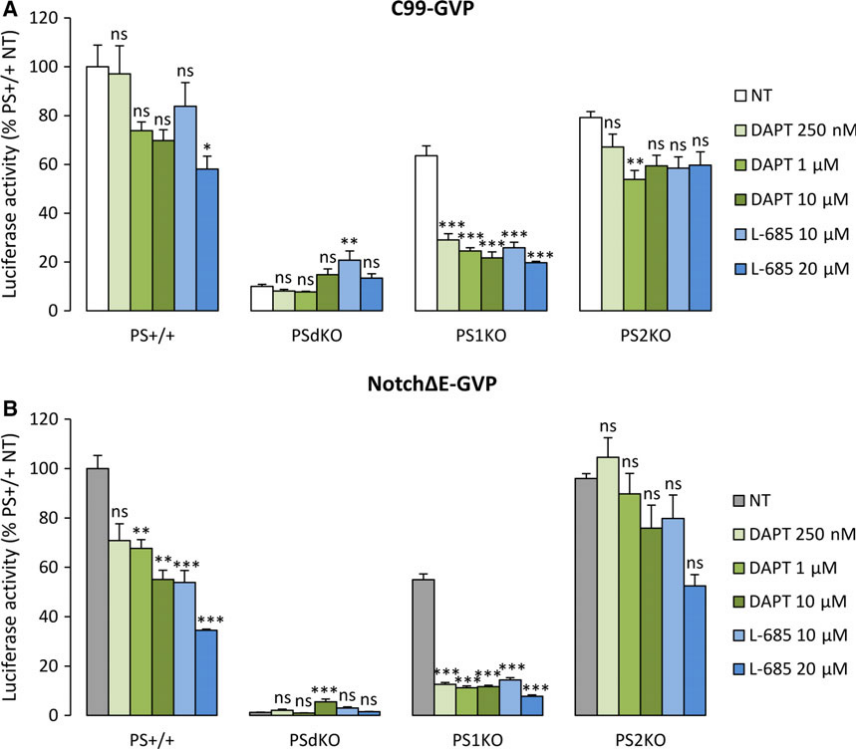
Effect of γ‐secretase inhibitors on AICD‐GVP and NICD‐GVP release in wild‐type, single and double‐knockout cell lines. (**A**) MEFs PS+/+, PSdKO, PS1KO and PS2KO were cotransfected with C99‐GVP construct and reporter genes and were treated for 16 hrs with DAPT 250 nM, 1 μM, 10 μM or L‐685 10 μM, 20 μM. (**B**) Wild‐type and knockout MEFs were cotransfected with NotchΔE‐GVP construct and reporter genes and were treated as described in (**A**). Quantification of AICD‐GVP (**A**) and NICD‐GVP (**B**) release was performed by measurement of luciferase activity. Results (means ± S.E.M.) are represented as percentages of signal measured in PS+/+ non‐treated (NT) cells. *P < 0.05, **P < 0.01, ***P < 0.001 *versus* corresponding NT cell type in each condition, *n* = 9 in three independent experiments.

We used these different cell lines to measure the effects of two well‐documented and specific γ‐secretase inhibitors that bind the active complex: DAPT 19 and L‐685,458 (noted here L‐685) 25. We first treated MEFs PS+/+, single and double‐KO cells with increasing concentrations of the two inhibitors, and measured the cleavage of C99‐GVP and Notch∆E‐GVP under these conditions (Fig. 3A and B). Treatments with both inhibitors repressed C99‐GVP processing in a dose‐dependent manner in PS+/+ cells. To note, they were even more efficient towards Notch∆E‐GVP processing. No inhibition was measured in PSdKO cells, confirming the specific action of the treatments on PSs‐dependent γ‐secretase activity. Results obtained in the single‐KO cells showed that γ‐secretase inhibitors were much more efficient on endogenous murine PS2 than PS1, as measured in PS1KO *versus* PS2KO cells, respectively. Maximal inhibition of C99‐GVP cleavage reached a plateau for both inhibitors at concentrations around 10‐20 μM in PS2‐expressing cells. A much lower and globally non‐significant inhibition (25%) was measured in PS1‐expressing cells (all these values are summarized in Table S1). A similar pattern was observed for Notch∆E‐GVP processing, with even greater inhibitory effects in PS2‐expressing cells at high concentrations (around 80%).

**Figure 4:**
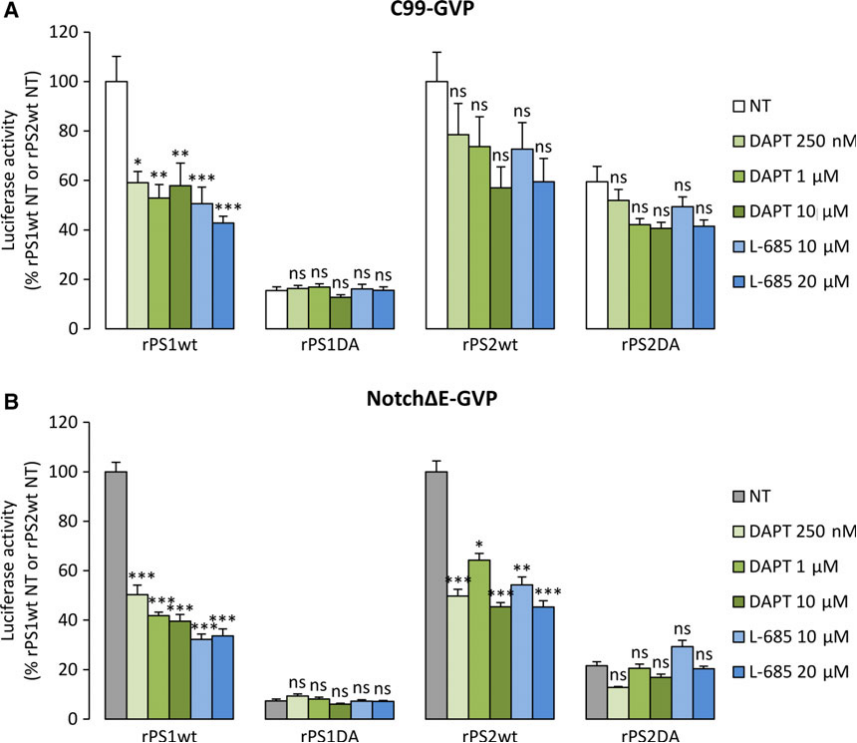
Effect of γ‐secretase inhibitors on AICD‐GVP and NICD‐GVP release in rescued cells expressing human wild‐type or mutated PS1 or PS2. (**A**) MEFs PSdKO rescued with PS1/PS2 wt/DA were cotransfected with C99‐GVP construct and reporter genes and were treated for 16 hrs with DAPT 250 nM, 1 μM, 10 μM or L‐685 10 μM, 20 μM. (**B**) Rescued cell lines were cotransfected with NotchΔE‐GVP construct and reporter genes and were treated as described in (**A**). Quantification of AICD‐GVP (**A**) and NICD‐GVP (**B**) release was performed by measurement of luciferase activity. Results (means ± S.E.M.) are represented as percentages of signals measured in non‐treated (NT) rPS1wt or rPS2wt cells, respectively. *P < 0.05, **P < 0.01, ***P < 0.001 *versus* corresponding NT cell type, *n* = 9 in three independent experiments.

Same experiments were carried out in PSdKO cells re‐expressing the wild‐type or catalytically inactive human PS1 or PS2 (Fig. 4A and B). Strikingly, the γ‐secretase inhibitors were efficient in rPS1wt cells, blocking both C99‐GVP and Notch∆E‐GVP processing, with maximal inhibitory effects of 57% and 68%, respectively. They significantly blocked Notch∆E‐GVP cleavage in rPS2wt cells (55% of inhibition at the highest concentrations), but were much less efficient on C99‐GVP processing (see Table S1), even if an inhibitory trend was observed. As expected, the γ‐secretase inhibitors were inactive in cells expressing rPS1DA and rPS2DA mutants.

All these results were obtained on the processing of exogenous APP and Notch substrates. We also addressed the effects of DAPT and L‐685 on endogenous APP and Notch (Fig. S2). Treatments with DAPT and L‐685 triggered the accumulation of the endogenous C‐terminal fragments of APP and Notch, due to inhibition of their processing by γ‐secretase. In PS1KO cells, we observed a noticeable accumulation of CTFs upon treatment, especially for APP‐CTFs. PS2KO cells exhibited no apparent sensitivity to inhibitors, except for DAPT on APP cleavage (Fig. S2A). In rescued cell lines, both inhibitors exerted an effect on cleavages of APP and Notch in rPS1 cells, while in rPS2 cells, the inhibitors showed no apparent effect on the accumulation of C‐terminal fragments (Fig. S2B). Overall, inhibitory treatments seemed to reveal comparable effects on exogenous *versus* endogenous substrates, depending on the nature of PSs they are targeting (murine or human). Importantly, this is not due to an altered subcellular distribution of rescued PSs *versus* endogenous ones, as shown in Fig. S3.

## Discussion

The physiological and pharmacological properties of the different γ‐secretase complexes are poorly understood, although several lines of evidence indicate that they might be differentially distributed in tissues and differentially involved in substrate processing 15, 16, 26. Different γ‐secretase subunits assemble in at least four human and six rodent complexes, differing by the presence of PS1 or PS2 and Aph1A or Aph1B (or Aph1C in rodents) with repercussions on the complex's properties. For instance, the biochemical and physiological consequences of genetically reduced γ‐secretase activity in different Aph1KO models were reported to be divergent and tissue specific 2. More recently, PS1 and PS2 were reported to target γ‐secretase complexes in distinct cellular compartments 15, 16. The localization of the PS1 or PS2 γ‐secretase complex determines substrate specificity, at least for APP processing 15. These very fundamental observations raise the following points that we addressed in the present study: (*i*) are PS1 and PS2 γ‐secretases differentially involved in the cleavage of their two major substrates, APP and Notch and (*ii*) do pharmacological inhibitors differentially target PS1 and PS2 γ‐secretases. We dissected the contribution of murine and human PS1 γ‐secretases in APP and Notch processing, and strikingly, we found that well‐characterized γ‐secretase inhibitors differentially target human and murine γ‐secretases.

In our study, we compared the γ‐processing of APP and Notch using a sensitive transactivation assay that measures the release of AICD and NICD, respectively 17. In this set‐up, AICD and NICD harbour a Gal4‐VP16 fusion sequence that allows a very sensitive and specific detection of AICD and NICD production by a Gal4 transactivation assay. The tagging of the APP and Notch proteins was previously shown not to affect their processing by γ‐secretase. The big advantage of this assay is to allow a direct comparison of the two cleavages in a homologous system, when compared to other reporter gene assays developed to measure APP and Notch processing independently 18.

To address the exact contribution of PS1 and PS2 γ‐secretases to substrate processing, we used PS(s) KO MEFs cells 27. Most of the cell types, including neurons, express both PS1 and PS2 26, making impossible to analyse the respective contribution of PS1 and PS2 in γ‐secretase activity measured in cells. In addition, the PSdKO MEFs cells allowed us to generate cell lines stably expressing different forms of human PS1 and PS2 proteins in a PS‐null background. *In vitro* γ‐secretase assays reported in our previous studies 18 indicate that γ‐secretase activity was functionally restored in these cells. This is confirmed here by the presence of activated PSs in rescued cells, attested by the presence of the PSs CTFs generated by endoproteolysis in the activation step of the γ‐secretase 28. These cellular models allowed us to analyse the catalytic properties of human and murine γ‐secretases differing only by the PSs subunit. This cannot be achieved by overexpression studies, as only a small pool of the overexpressed exogenous PSs is activated and incorporated in active γ‐secretases 29.

We found that γ‐secretase activity is finely tuned in cells and has to be kept in a defined range, as deficiency in one PS is compensated by an increased expression of the other. PS2 overexpression was already shown to down‐regulate PS1 expression 24. Here, we found that the absence of PS1 strongly up‐regulates PS2 at the transcriptional level. This could suggest in another way that transcription of PS2 is down‐regulated by PS1 in physiological conditions. The significance of this observation in cell physiology and the underlying mechanisms of PS1‐dependent transcriptional regulation of PS2 are unknown and await further investigation. Still, it raised an important point for the interpretation of our study. Indeed, our single PSKO MEFs cells are very useful to analyse and compare the enzymatic properties of PS1 and PS2 γ‐secretases, but conclusions about the respective contribution of these γ‐secretases to substrate processing can hardly be extrapolated.

Several studies have reported a lower activity of PS2 γ‐secretases compared to PS1 γ‐secretases 16, 30, 31. Our work confirms, both in single‐KO and rescued cells, that APP substrates are more efficiently processed by PS1 than by PS2 and this is in line with published results showing a predominant implication of PS1 in the γ‐secretase activity 21, 22, 32, 33. However, we observed that PS2‐expressing cells still efficiently cleave C99‐GVP. This was also previously reported 34 and raises the hypothesis that lower cleavage of APP by PS2 γ‐secretase could be related to a reduced availability of APP‐CTFs for PS2 γ‐secretase, rather than a poor affinity. Indeed, PS2 γ‐secretases are enriched in late endosomes and lysosomes 15, and APP might be efficiently processed in cells along trafficking through the secretory pathway and at the plasma membrane 16 before reaching endosomal compartments.

In PSdKO cells, we found the background signal in C99‐GVP expressing cells to be higher than the Notch∆E‐GVP signal. Although very low (10% of the signal measured in PS+/+ cells), this would suggest that a PS‐independent γ‐secretase activity towards C99‐GVP exists in these cells. Previous reports indicated the possible existence of PS‐independent γ‐secretase activity, either by *in vitro* γ‐secretase assay or by measurement of Aβ production in PSdKO cells 35, 36. PS‐independent γ‐secretase activity has been a matter of controversy 37 but it could suggest that the basal activity of C99‐GVP observed in PSdKO results from a processing not operated by PSs. However, the C99‐GVP construct was previously tested in PSs deficient cells (PSdKO mouse blastocyst‐derived BD8 cell line) and did not exhibit any signal 17. Thus, the signal measured in PSdKO cells is more likely to result from leaking of a small portion of non‐cleaved C99‐GVP to the nucleus, rather than from a proteolysis of C99‐GVP.

One important asset of our model is to overcome biases encountered by simple PSs overexpression 38, 39, 40. The major biases are abundant levels of inactive PSs and mislocalization of PSs in cells. Here, the rescued cell lines showed no accumulation of inactive (full‐length) PSs and, importantly, a subcellular localization of PSs in line with recent studies 15, 16 showing different cellular distribution of PS1 and PS2 γ‐secretases. In addition, no differences were observed between endogenous (murine) and rescued (human) PS1 or PS2 distributions (Fig. S1).

Regarding PS1 and PS2 activities, we made an unexpected observation. We detected a residual γ‐cleavage of C99‐GVP with the catalytically inactive PS2DA mutant. This was not observed with the PS1DA mutant. No active endoproteolysed hPS2 was detected in the rPS2DA cell line. One hypothesis could be that the presence of non‐cleaved PS2 favours an unspecific transactivation by C99‐GVP (*e.g*. without γ‐cleavage, or by cleavage by an unknown protease). Alternatively, studies have reported the formation of complexes between APP and PS2, but not PS1, in transfected cells 41, indicating specific interactions between APP and PS2 that could explain this observation. Finally, full‐length PS2 could integrate the γ‐secretase complex and display partial γ‐secretase activity. This last point has never been illustrated so far and is purely hypothetical, and *in vitro* assays did not show a restoration of γ‐secretase activity by rPS2DA mutants 18, strongly suggesting that the effects observed with rPS2DA cells here might be independent of C99‐GVP cleavage.

Our most striking observation is that specific and well‐described inhibitors differentially inhibited γ‐secretase complexes containing either endogenous (murine) or rescued (human) PSs. DAPT 19 was found to specifically bind to PS1 (not PS2) CTFs in human HeLa cells, to a domain distinct from the catalytic site or substrate binding site 42. L‐685 is a transition state analogue inhibitor 25 directed to the active site of the γ‐secretase, that binds to PS1 and PS2 proteolytic fragments from human HeLa cells and mouse N2a neuroblastoma 43. We found that DAPT efficiently blocks the cleavage of C99‐GVP and NotchΔE‐GVP in cells expressing human PS1, in agreement with the study of Dovey and collaborators 19. However, DAPT only efficiently blocked the processing of NotchΔE‐GVP in cells expressing human PS2, indicating that even at sub‐micromolar concentrations (*i*) DAPT blocks PS2‐dependent γ‐secretase activity (*ii*) the inhibitory effects of DAPT on APP and Notch processing can be discriminated. The first observation might be in contradiction with data indicating that DAPT does not bind to PS2. We suggest here that DAPT efficiently inhibits and thus probably binds to human PS2, but might bind preferentially to human PS1 in cell extracts where both PS1 and PS2 are expressed. DAPT was found to inhibit more efficiently Notch processing than APP processing in human PS2‐expressing cells. To note, inhibition of Notch signalling by DAPT is documented 44. The same observations hold true for L‐685, a competitive γ‐secretase inhibitor. Thus, differential effects of inhibitors on APP and Notch processing do not depend on their inhibitory mechanism (competitive/non‐competitive), but rather on the presence of human PS1 or PS2 in the γ‐secretase complex. Strikingly, the inhibitory effects of DAPT and L‐685 were opposite on γ‐secretases containing murine PS1 or PS2 and γ‐secretases containing human PS1 or PS2. Both DAPT and L‐685 efficiently inhibited APP and Notch processing only in PS1KO MEFs cells, thus solely expressing endogenous PS2. They inhibited to a greater extent NotchΔE‐GVP than C99‐GVP cleavage (around 80% and 60%, respectively).

Most of these observations hold true for endogenous substrates (Fig. S2). In single‐knockout cell lines, inhibitors were able to block efficiently the murine PS2‐dependent processing of APP‐CTF, and slightly the one of NotchΔE. In PS2KO cells, excepted DAPT inhibition of APP‐CTF processing that has also been observed as significant at 1 μM concentration on C99‐GVP, both inhibitors appeared to be poorly efficient on murine PS1‐dependent activities. In rescued cell lines, both DAPT and L‐685 distinctly inhibited human PS1‐dependent processing to a greater extent that human PS2‐dependent processing, for both APP‐CTF and NotchΔE.

The different pharmacological properties of γ‐secretase containing murine or human PSs have not been reported so far to our best of knowledge. They could result from intrinsic properties (domains, structure and folding) that are different between human and rodent PSs. One other hypothesis could be that the catalytic properties of rodent γ‐secretases differ from human γ‐secretases because of differences in the structure and composition of the subunits. Indeed, rodent can express six different γ‐secretases, whereas only four human γ‐secretases can be assembled. Apart from PS1 and PS2, human cells express two Aph1 subunits (A and B), whereas the duplication of the Aph1B gene in rodents gives rise to a third Aph1 protein (Aph1C). A very recent study indicated that trafficking of the γ‐secretase complex depends on the Aph1 variant expressed in the cells 16. Thus, catalytic properties of the γ‐secretase and specificity towards the processing of a particular substrate depend on both its subunit composition and subcellular localization. This is confirmed by our results, showing differential effects of pharmacological inhibitors on γ‐secretase containing either human or murine PS1 and PS2. But very importantly, it also indicates that results of γ‐secretase inhibitors tested in mice models cannot be directly extrapolated to human γ‐secretases. This point is critical for trials evaluating the therapeutic interest of small molecules targeting the γ‐secretase, which have been unsuccessful so far.

## Author contributions

S.S. and P.K.C. designed the research study; S.S., C.V. and B.T. conducted experiments; C.M. and H.K. built MEFs rescued cells and luciferase constructs; S.S., C.V. and P.K.C. analysed the data; S.S., C.V. and P.K.C. wrote the article; all the authors have read and approved the final manuscript.

## Conflict of interest

The authors confirm that there are no conflict of interests.

## Supporting information


**Figure S1** Expression of C99‐GVP and NotchΔE‐GVP in PS+/+ transfected cells.

**Figure S2** Effect of γ‐secretase inhibitors on endogenous APP and Notch CTFs accumulation.

**Figure S3** Immunocytochemical characterization of PS1 and PS2 subcellular localization in knockout and rescued models.

**Table S1** Details of DAPT and L‐685 inhibitory effect on murine and human PSs cleavage activity in MEFs cells transfected with exogenous substrates.
