# Supplementary material for: Specificity of presenilin‐1‐ and presenilin‐2‐dependent γ‐secretases towards substrate processing
Source: J Cell Mol Med. 2017 Oct 10;22(2):823–33. doi: 10.1111/jcmm.13364 (PMC5783875; doi:10.1111/jcmm.13364)
Supplement: Supplementary file 1 — Figure S1 Expression of C99‐GVP and NotchΔE‐GVP in PS+/+ transfected cells. [file JCMM-22-823-s001.pdf]

Supplementary Figure S1

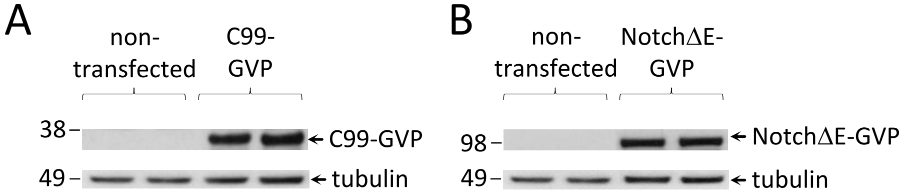

Supplementary FigS1. **Expression of C99-GVP and NotchΔE-GVP in PS+/+ transfected cells**. Cell lysates were analyzed by Western blotting using an antibody directed against the VP16 domain of the GVP fusion protein. Tubulin served as a loading control. Cells transfected with either C99-GVP (A) or NotchΔE-GVP (B) exhibit bands at the expected molecular weights (indicated by arrows), which are absent in non-transfected cells.
