# Supplementary material for: Specificity of presenilin‐1‐ and presenilin‐2‐dependent γ‐secretases towards substrate processing
Source: J Cell Mol Med. 2017 Oct 10;22(2):823–33. doi: 10.1111/jcmm.13364 (PMC5783875; doi:10.1111/jcmm.13364)
Supplement: Supplementary file 2 — Figure S2 Effect of γ‐secretase inhibitors on endogenous APP and Notch CTFs accumulation. [file JCMM-22-823-s002.pdf]

Supplementary Figure S2

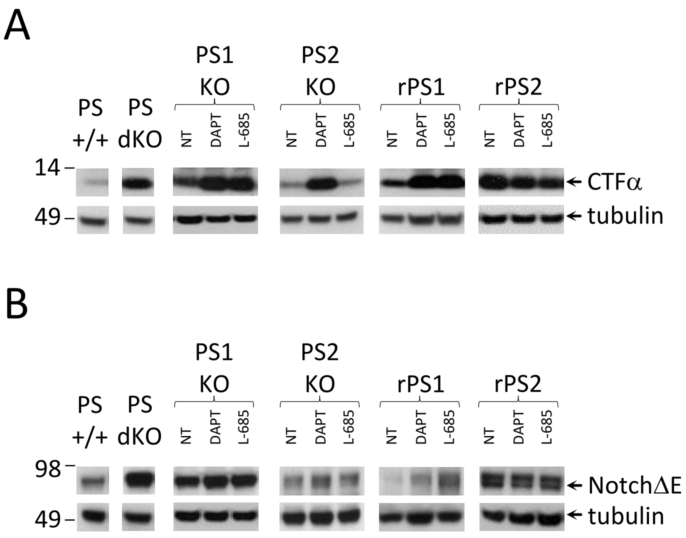

Supplementary FigS2. **Effect of γ-secretase inhibitors on endogenous APP and Notch CTFs accumulation**. All MEFs were plated in 6-well plates and treated 24h after seeding with either DAPT 10μM or L-685 10μM. Cells were harvested 16h after treatment for Western blotting. PS+/+ and PSdKO were used as positive and negative controls of substrates cleavage, respectively. APP-CTFα (A) and NotchΔE (B) were detected using specific antibodies targeting the C-terminal regions of the full-length APP and Notch proteins. Expected positions of APP CTFα and NotchΔE are indicated by arrows. Tubulin served as a loading control.
