# Supplementary material for: Specificity of presenilin‐1‐ and presenilin‐2‐dependent γ‐secretases towards substrate processing
Source: J Cell Mol Med. 2017 Oct 10;22(2):823–33. doi: 10.1111/jcmm.13364 (PMC5783875; doi:10.1111/jcmm.13364)
Supplement: Supplementary file 3 — Figure S3 Immunocytochemical characterization of PS1 and PS2 subcellular localization in knockout and rescued models. [file JCMM-22-823-s003.pdf]

Supplementary Figure S3

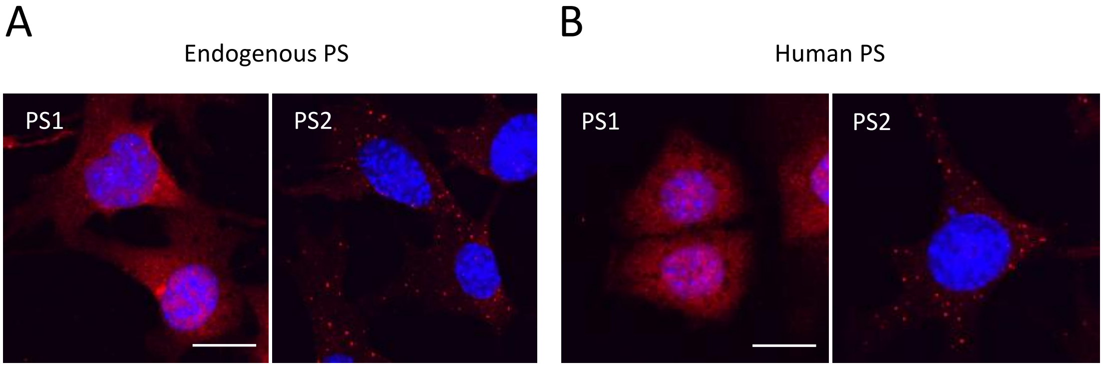

Supplementary FigS3. **Immunocytochemical characterization of PS1 and PS2 subcellular localization in knockout and rescued models**. Cells were fixed with 4% paraformaldehyde (PFA) in PBS and immuno-stained for PS1 or PS2 (red) and DAPI (blue). Stainings have been analyzed by confocal microscopy to generate *z*-stack images. Endogenously expressed PSs (A) and human PSs isoforms stably re-expressed by lentiviral infection (B) show an identical distribution in the cell. Scale: 20µm.
