# Supplementary material for: Specificity of presenilin‐1‐ and presenilin‐2‐dependent γ‐secretases towards substrate processing
Source: J Cell Mol Med. 2017 Oct 10;22(2):823–33. doi: 10.1111/jcmm.13364 (PMC5783875; doi:10.1111/jcmm.13364)
Supplement: Supplementary file 4 — Table S1 Details of DAPT and L‐685 inhibitory effect on murine and human PSs cleavage activity in MEFs cells transfected with exogenous substrates. [file JCMM-22-823-s004.doc]

**Supplementary Table1. Details of DAPT and L-685 inhibitory effect on murine and human PSs cleavage activity in MEFs cells transfected with exogenous substrates.** Cells were transfected with either C99-GVP or NotchΔE-GVP construct and with reporter genes, were treated with DAPT 250nM, 1µM, 10µM or L-685 10µM, 20µM for 16h and were quantified for AICD-GVP or NICD-GVP production using the Dual-Glo® Luciferase Assay System. NI stands for “no inhibition”. *p<0.05, **p<0.01, ***p<0.001 vs corresponding NT cell type, n=9 in 3 independent experiments.

|  |  | **PS+/+** | **PSdKO** | **PS1KO** | **PS2KO** | **rPS1wt** | **rPS1DA** | **rPS2wt** | **rPS2DA** |
| --- | --- | --- | --- | --- | --- | --- | --- | --- | --- |
| **C99-GVP** | DAPT 250nM | 2.86 | 19.31 | 54.30*** | 15.25 | 40.92* | NI | 21.46 | 12.65 |
|  | DAPT 1µM | 26.19 | 22.84 | 61.40*** | 31.93** | 47.10** | NI | 26.29 | 29.04 |
|  | DAPT 10µM | 30.24 | NI | 65.85*** | 24.94 | 42.15** | 17.86 | 43.01 | 31.58 |
|  | L-685 10µM | 16.21 | NI | 59.24*** | 26.11 | 49.39*** | NI | 27.32 | 16.88 |
|  | L-685 20µM | 41.92* | NI | 68.94*** | 24.61 | 57.25*** | NI | 40.50 | 30.27 |
| **NotchΔE-GVP** | DAPT 250nM | 29.13 | NI | 77.08*** | NI | 49.72*** | NI | 50.28*** | 40.53 |
|  | DAPT 1µM | 32.32** | 22.77 | 79.60*** | 6.48 | 58.19*** | NI | 35.70* | 4.77 |
|  | DAPT 10µM | 44.90** | NI | 78.82*** | 20.91 | 60.40*** | 17.79 | 54.62*** | 22.12 |
|  | L-685 10µM | 46.12*** | NI | 73.92*** | 16.87 | 67.82*** | 1.72 | 45.71** | NI |
|  | L-685 20µM | 65.46*** | NI | 85.91*** | 45.30 | 66.34*** | 2.38 | 54.70*** | 5.73 |
